# Assessing *De Novo* transcriptome assembly metrics for consistency and utility

**DOI:** 10.1186/1471-2164-14-465

**Published:** 2013-07-09

**Authors:** Shawn T O’Neil, Scott J Emrich

**Affiliations:** 1 Center for Genome Research and Biocomputing, Oregon State University, Corvallis, OR 97333, USA; 2 Department of Computer Science and Engineering, University of Notre Dame, Notre Dame, IN 46556, USA

## Abstract

**Background:**

Transcriptome sequencing and assembly represent a great resource for the study of non-model species, and many metrics have been used to evaluate and compare these assemblies. Unfortunately, it is still unclear which of these metrics accurately reflect assembly quality.

**Results:**

We simulated sequencing transcripts of *Drosophila melanogaster*. By assembling these simulated reads using both a “perfect” and a modern transcriptome assembler while varying read length and sequencing depth, we evaluated quality metrics to determine whether they 1) revealed perfect assemblies to be of higher quality, and 2) revealed perfect assemblies to be more complete as data quantity increased.

Several commonly used metrics were not consistent with these expectations, including average contig coverage and length, though they became consistent when singletons were included in the analysis. We found several annotation-based metrics to be consistent and informative, including contig reciprocal best hit count and contig unique annotation count. Finally, we evaluated a number of novel metrics such as reverse annotation count, contig collapse factor, and the ortholog hit ratio, discovering that each assess assembly quality in unique ways.

**Conclusions:**

Although much attention has been given to transcriptome assembly, little research has focused on determining how best to evaluate assemblies, particularly in light of the variety of options available for read length and sequencing depth. Our results provide an important review of these metrics and give researchers tools to produce the highest quality transcriptome assemblies.

## Background

For non-model species with little or no available genomic resources, transcriptome sequencing offers a cost-effective method of characterizing the gene set for a species of interest. Sequencing of Expressed Sequence Tags (ESTs) can quickly and cheaply provide sequence data for a large percentage of expressed transcripts, which can then be assembled into longer transcript-representative sequences. These can then be annotated for functionality (e.g. [1,2]), assessed for genetic diversity [3,4], and, if technology and sequencing depth allow, simultaneously provide a snapshot of gene expression at the time of sequencing [5,6]. These data can also provide the basis for microarray (or RNA-seq) designs that determine expression differences in experimental contexts (e.g. [7]). These applications, however, often require complete and accurate assemblies of EST sequences.

Transcriptome assembly efforts can be challenging, though, for biological and technical reasons. Since traditional Sanger sequencing was used to sample ESTs, several newer generation sequencing platforms provide varied options for read length and sequencing depth—both important considerations as transcripts can vary in size and abundance. Assemblers used in recent transcriptome projects include SeqMan [1,8], MIRA [9,10], Celera Assembler [4], CAP3 [2,4,8,10], and Newbler [3,5,11-14], as well as de-Bruijn graph based assemblers such as Velvet, Oases [6] and NGEN [15]. Comparisons suggest that assemblers capable of accounting for alternative splicing perform best [8,13,16-18].

While these comparisons between assemblers (or assemblies) use common quality metrics, it is unclear how accurate the metrics themselves are. Many metrics such as singleton and contig count, coverage, N50, and overall assembly size were simply repurposed from whole-genome assembly evaluation. For example, N50 contig length is traditionally considered best when maximized and has been used to assess transcriptome assembly quality [8,14,19]; however, this metric should not exceed the true transcript N50 length that is usually unknown *a priori*. Similarly, average or median coverage statistics may relate to sequencing depth, but non-uniform expression may make comparisons between them less informative than for genome assemblies [20-22].

Annotation-based metrics represent an alternative assessment tool for transcriptome projects. After matching assembled sequences to protein sequences of a related organism, statistics such as the number of unique proteins and average percentage of protein sequences matched are reported [4,13,19]. Associating assembled sequences with homologous proteins in this way provides a biologically relevant perspective including redundancy of assembled sequences, diversity of functions represented, and completeness [4]. These metrics, however, depend on the evolutionary distance of the comparison, and for non-model species often require annotation against distant relatives where orthologs may have been duplicated, lost, or undergone changes in size.

### Assessing quality metrics

While it is important to understand how different assembly methods and sequencing technologies perform when processing transcriptome data, we should also understand how these metrics relate to properties of the assembly process. As the quality of a transcriptome assembly improves (either through more or better input data, or better assembly methods) a good metric will reflect this change in a strong and identifiable manner.

Assessing a metric for assembly quality requires objective standards of measurement, which we produce via simulating sequencing of a well understood transcript dataset from *Drosophila melanogaster* (see Methods for details). Because we simulate sequencing, we know the source location of each read and can simulate a “perfect” assembly that correctly detects all overlaps and can correct all sequencing errors. Simultaneously, we can assemble the simulated reads using a (non-perfect) software assembler (Newbler, recent versions of which attempt to assemble alternative isoforms [13]). Processing the data in this way while varying both the simulated sequencing depth and average read length, we may assume that: 

1. For a given dataset, the perfect assembly will be of higher quality than the non-perfect assembly.

2. For the perfect assemblies, increasing sequencing depth and average read length will result in more complete assemblies.

Note that fact two may not hold for any non-perfect assembler as assumptions concerning the expression distribution or read length may degrade results even in the presence of increased data quantity.

Any good metric for transcriptome assembly quality should be consistent with these two facts. If a metric reveals perfect assemblies to be of higher quality over all sequencing depths or read lengths, we say it is consistent over sequencing depth or read length. Further, if the metric trends in a similar monotonic way over increased sequencing depth as well as read length for perfect assemblies (and thus allows for unambiguous comparisons for all tested cases) we say it is “fully consistent.”

While several studies have compared assembler performance for a variety of datasets (e.g. [4,8,15,23]) only Mundry et al. have directly evaluated the quality metrics themselves [18] using a similar simulation-based approach as outlined here but without varying sequencing depth or read length. Studying the effects of read count and read length on assembly metrics not only provides another dimension for metric comparison, but is also informative as these are two variables largely under researchers’ control.

We note that this comparison between perfect assemblies and those produced by a commonly used assembler are not meant specifically to highlight deficiencies in the non-perfect assembly process, nor are the simulated sequencing choices meant to favor one method or sequencing technology. Indeed, given the complexity, redundancy, and error characteristics of transcript sequences, no existing method could be expected to produce results on par with perfect assemblies. Instead, it is precisely this necessary deficiency that we use to better understand metrics for transcriptome assembly quality.

## Results

### Simulated sequencing

We simulated sequencing from the *Drosophila melanogaster* transcript set (FlyBase release 5.38), which includes known alternate splice forms (of 13,918 genes, 4,263 are alternatively spliced for a total of 23,711 characterized transcripts) as well as untranslated regions that would be sequenced by a cDNA strategy (see Methods for details). Evidence suggests that true gene expression distributions are complex [21]; however, it has been recognized for some time that a power-law distribution with exponent -1 provides an approximation [20]. Thus, we randomly ordered transcripts, and sampled each transcript in position *i* with relative probability 1/*i*. Sampling rate was also proportional to length, with longer transcripts having higher probability of selection (based on a random fragmentation model; see Methods).

As an initial scenario, we first simulated sequencing of reads of average length 400 bp (normally distributed with standard deviation of 100 bp and minimum length of 100 bp provided by resampling) and varied read numbers as follows: 100K, 200K, 400K, 600K, 1M, 1.4M, 1.8M, and 2.2M. Similarly, holding read number constant at 600K, we varied the average read length: 200 bp, 400 bp, 600 bp, 800 bp, and 1000 bp. In these cases, standard deviation and minimum of read length were set to 25% of average length. All reads were sequenced with a 1.5% uniform error rate (that does not affect any perfect assemblies; see Methods). Because sequencing technologies continue to evolve, these parameters are not chosen to represent any particular technology, but rather to sample a range of read lengths likely to be prevalent in the near future (see e.g. [24,25]). While for simulation and assembly efficiency the depths of sequencing we considered are generally lower than for current-generation technologies, we note that our non-normalized 2.2 million simulated read dataset represents over 98% of the *D. melanogaster* transcript set (see section ‘Annotation and rarefaction’), supporting the use of aggressive dataset pruning [19].

### Assembly statistics

The most basic metrics for transcriptome assemblies are aggregate and concern the size of the output. These include assembly size (in base pairs), percentage of reads assembled into contigs, and counts of contigs and singletons.

The notions of contig and singleton are straightforward for perfect assemblies: a contig is any sequence produced by two or more overlapping reads, while singletons are the remaining isolated reads. By contrast, the assembler we compare with produces a variety of output types: first, portions of overlapping reads are assembled into “contigs” representing putative exons. Groups of contigs that appear to constitute a single gene are then arranged to form “isotigs” representing putative splice variants of the gene. Note that an isotig may consist of only a single contig. When this splice variant reconstruction fails, some “orphan” contigs may be unused in isotigs. Thus, unique sequence in a Newbler assembly is represented by unassembled singleton reads, (orphan) contigs, and isotigs. For our purposes we consider both Newbler orphan contigs and isotigs as unique assembled sequence comparable to perfectly assembled contigs. We shall refer to this combined set of orphan contigs and isotigs as *c-isotigs*. Further, we shall refer to the combined set of perfect contigs and singletons (and non-perfect c-isotigs and singletons) for a single assembly as the set of *unigenes*.

**Figure 1:**
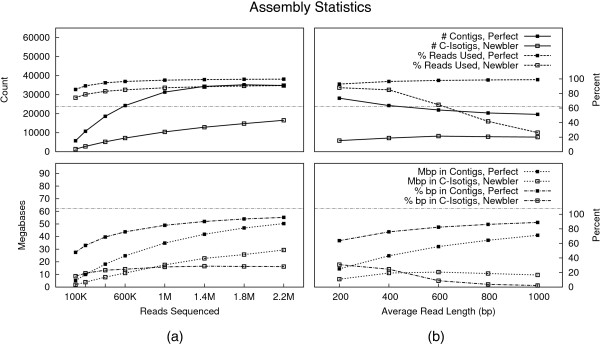
**Basic assembly statistics.** Counts of assembled contigs and c-isotigs, percents of reads incorporated in contigs and c-isotigs, aggregate contig and c-isotig sequence sizes, and percentage of total assembly output represented in contigs and c-isotigs as sequencing depth **(a)** and average read length **(b)** varies. True transcript count and transcriptome size are shown as dashed horizontal lines for reference.

Figure 1(a) shows contig and c-isotig counts as well as percent of reads incorporated into the assemblies as sequencing depth increases. Figure 1(a) also shows the aggregate size of the contigs and c-isotigs in bases, as well as the percentage of the total assemblies (including singletons) present in assembled pieces. Reference lines indicate the true number of transcripts and transcriptome size.

As expected for the perfect assemblies, as read coverage increased, the percentage of reads incorporated into the assembly grew to near 100%. Further, the percentage of unigene bases present in contigs also grew to near 100%. Although the non-perfect assembler incorporated an increasing percentage of reads in c-isotigs as sequencing depth grew, this percentage lagged that of the perfect assemblies and only reached 90% at the highest sequencing depths. As a consequence, the percentage of unigene bases in c-isotigs was small, and actually reduced slightly as the number of reads sequenced exceeded 1.4M.

For both assembly types, the total size of assembled sequence grew to approach the true transcriptome size of 61 Mbp, though the total size of perfectly assembled contigs was nearly twice that of assembled c-isotigs across the range of sequencing depths. While the number of c-isotigs strictly increased toward the correct number of transcripts from below, perfect contig count initially grew then decreased toward the true transcript number as contigs were joined.

Figure 1(b) and Table 1 show these same trends as read number was held at 600K and average read length was varied. Results concerning percent of reads used and percent of total bases in contigs were similar for the perfect assemblies. Contig count for the perfect assembly began slightly above the true transcript count, and decreased slightly as improved read lengths joined contigs.

**Table 1:** Metric trends and consistency

**Metric**	**Trend by**	**Consistent over**	**Trend by**	**Consistent over**	**Fully consistent**
	**sequencing depth**	**sequencing depths**	**read length**	**read lengths**	**metric**
Contig count		*✓*		*✓*	✗
% of reads used in contigs		*✓*		*✓*	*✓*
BP in contigs		*✓*		*✓*	*✓*
% BP in contigs		*✓*		*✓*	*✓*
Average contig coverage		✗		✗	✗
Average unigene coverage		*✓*		*✓*	*✓*
Contig read count COV		*✓*		*✓*	✗
Unigene read count COV		*✓*		*✓*	✗
Average contig length		✗		✗	✗
Average unigene length		*✓*		*✓*	*✓*
Contig N50 length		✗		✗	✗
Unigene N50 length		*✓*		*✓*	*✓*
Unique annotations in singletons		*✓*		*✓*	✗
Unique annotations in contigs		*✓*		*✓*	*✓*
Unique annotations in unigenes		✗		*✓*	✗
Average contig OHR		✗		✗	✗
Average unigene OHR		*✓*		*✓*	*✓*
Contig RBH count		*✓*		*✓*	*✓*
Unigene RBH count		*✓*		*✓*	*✓*
% of Annotated contigs with RBHs		✗		✗	✗
% of Annotated unigenes with RBHs		*✓*		✗	✗
Average contig CF		*✓*		*✓*	*✓*
Average unigene CF		✗		✗	✗
Unique reverse annotations in contigs		*✓*		*✓*	*✓*
Unique reverse annotations in unigenes		*✓*		*✓*	*✓*

Trends for *de novo* transcriptome assembly metrics as sequencing depth and read length are varied. Straight lines indicate monotonically increasing or decreasing trends, while curved lines indicate non-monotonic trends (either increasing then decreasing, or decreasing then increasing). Metrics are considered consistent if they consistently ranked perfectly assemblies as better; fully consistent metrics are consistent metrics with similar monotonic trends by sequencing depth and read length for perfect assemblies. Annotation-based metrics shown are computed in comparison to *B. mori* proteins. See Additional file 1 for full results.

Results for the realistic assemblies showed a reversed trend as read length increased. Above a 400 bp average read length, the percentage of reads included in the assembly process decreased until at 1,000 bp only 25% of reads were assembled together. Further, at the 1,000 bp read length, less than 2% of unigene bases were present in c-isotigs reflecting very large total outputs consisting almost entirely of singletons.

Overall, statistics for the realistic 1.8M read/400 bp assembly were similar to those reported by Ewen-Campen et al. who used Newbler to assemble ≈2 million reads of median length 300 bp (total assembled bases [Ewen-Campen et al.]: 25 Mbp [20 Mbp], singletons: 173K [168K], isotigs: 14.3K [20.9K] [13]).

**Figure 2:**
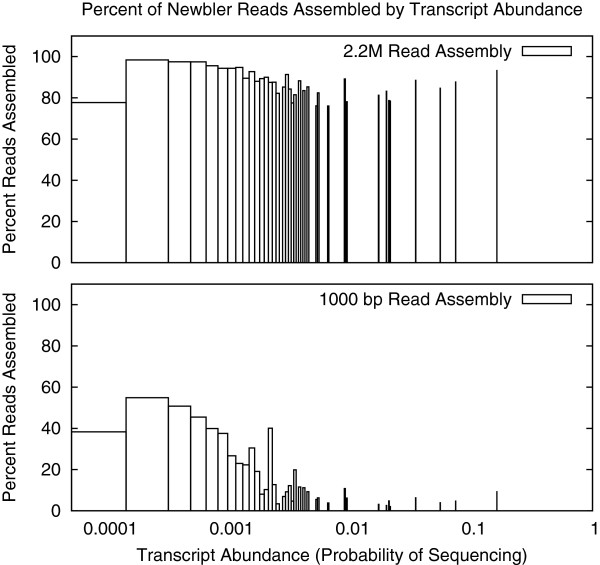
**Newbler read usage by transcript sampling rate.** Percentage of reads assembled, binned by transcript sequence abundance, for the 2.2M read and 1,000 bp read length Newbler assemblies. Fewer reads were assembled for transcripts with high abundance, particularly for the long read dataset.

Ewen-Campen et al. reported their singletons to be highly redundant based on annotation, and employed a secondary CAP3 assembly strategy for them. To assess the redundancy of singletons produced by the non-perfect assembler, we compared singleton counts by source transcript to the simulated sampling frequency. We found that for both the highest sequencing depth and longest read length assemblies, most singletons were sourced from transcripts with the highest representation. Figure 2 shows average read usage for transcripts in these assemblies binned by probability of read selection. In both cases, reads from the rarest transcripts were more likely to be left as singletons (as expected given our non-uniform sampling; see Methods). For the high sequencing depth assembly, read use initially decreases then increases slightly as transcripts become more abundant. For the long read length assembly, read usage is overall lower and drops significantly as abundance increases: only 4–10% of reads are assembled from the most common transcripts.

### Contig statistics

The next class of transcriptome assembly metrics concerns the intrinsic properties of the assembled sequences including length, coverage (number of bases incorporated in contigs from reads divided by length) and N50 length, defined as the shortest sequence length such that half of the total sequence output length is included in sequences that are shorter. These statistics are often computed only over the contig set (e.g. [8,15,18]), though we show below that including singletons, which frequently represent unassembled transcripts as discussed above, can significantly impact comparisons.

Table 1 shows the trends for contig and c-isotig coverage and length statistics as sequencing depth increases (full results for this section can be found in Additional file 1: Figure S1). As would be expected, both the length and coverage increased with increased sequencing depth for both assembly types. Because of the uneven sampling distribution, average coverage provided a stronger signal than median coverage—median coverage for the perfect (realistic) assemblies ranged from 1.6× to 2.5× (4.0× to 5.7×), while average coverage ranged from 3.0× to 9.3× (9.7× to 18.6×).

Average and N50 contig lengths approached the true values from below. For the perfect assemblies, at the highest sequencing depths length statistics are approximately half of those for the original transcript set. This, combined with Figure 1(a) showing that upwards of 50 Mbp of sequence is recovered in perfect contigs, indicates that increased sequencing would largely join existing contigs rather than discover new sequence, with the exception of very rare transcripts. For both assembly types, N50 lengths were longer than averages and followed similar trends.

Table 1 also shows trends for coefficients of variation (standard deviation divided by mean—a scale invariant measure of dispersion) of the number of reads used in contigs and c-isotigs as sequencing depth varies. For the perfect assemblies, this measure increased initially as the read usage distribution for contigs reflects the dramatic disparities in simulated transcript expression, then decreased slightly for the 22M read assembly as reads and contigs were joined to represent individual transcripts and rare transcripts were represented by contigs. By contrast, coefficients of variation for the realistic assemblies were much smaller and did not vary greatly across assemblies. This indicates that c-isotigs were erroneously uniform in read count, both within and between assemblies, and not as representative of the underlying expression distribution even for high sequencing depths.

Table 1 also shows contig and c-isotig trends as average read length was varied. Trends for the perfect assemblies were largely similar with the exception of contig read count coefficient of variation, which strictly decreases. These results reiterate the assembly difficulty experienced for reads longer than 400 bp—average c-isotig coverages, lengths, and N50 lengths decrease significantly as most reads are left out of the assemblies.

Comparison between perfect contigs and c-isotigs revealed that Newbler assemblies were on average longer and more highly covered. At first glance, these statistics suggest that these assemblies were of higher quality. When singletons were included and metrics were computed over all unigenes, however, we found that average length, coverage (considering singletons to have coverage of 1.0), and N50 lengths reflected the higher quality of perfect assemblies (Table 1, Figures S1(c) and S1(d)). For assemblies where singletons were dominant, however, the trends were not strong over read length or sequencing depth (with the exception of unigene N50 length as read length varied, Additional file 1: Figure S1(d)) providing little basis for comparison. Thus, it appears that while including singletons is necessary to make coverage and length statistics fully consistent metrics, doing so may result in metrics too invariant to be useful in practice.

### Annotation and rarefaction

Assembled transcriptomes are often compared to protein datasets of related, well characterized species in an effort to assess sequencing and assembly completeness in a biological sense. BLAST is usually used for this purpose with the best match as determined by minimum e-value (subject to some cutoff, which plays an important role in annotation rates [26], though cutoffs of ≈10^−6^ are commonly used, e.g. [13,27]) providing the annotation [22]. Although it is generally impossible to know how many transcripts are produced by an organism without a full genome sequence, annotation rates and percentage of proteins matched as a function of sequencing depth (rarefaction) are thought to speak to the percentage of all transcripts that are sequenced and assembled [1,11].

We annotated unigenes of each assembly using BLASTX with a 10^−6^ cutoff against two protein databases: *Bombyx mori* predicted proteins (GLEAN produced consensus gene set, SilkDB v2.0 [28]) and *Drosophila melanogaster* proteins (of the same release as the source transcript set: FlyBase v5.38). The latter represents a comparison of no evolutionary distance, which will be useful in studying the effects of annotation against related species just as perfect assemblies inform assembly metric comparisons. Because Newbler assemblies for average read lengths longer than 400 bp produced too many singletons (representing 840 Mbp in ≈1 million sequences), we annotated c-isotigs only for these assemblies.

Unique annotation counts for contigs and c-isotigs revealed increasing estimated discovery rates as sequencing depth increased (Table 1, Additional file 1: Figure S2(a)). For perfect assemblies, we also annotated contigs and singletons based on the transcripts that constituent reads were originally sourced from. For the perfect assemblies, this “true” annotation indicated that at high sequencing depths nearly 100% of transcripts were represented in contigs (Additional file 1: Figure S2(a)). However, because estimated discovery is based on unique annotations and many transcripts and domains are similar enough to produce erroneous annotation, BLAST-based annotation against the *D. melanogaster* protein dataset suggested only a 70% discovery rate for the 2.2M read assembly. Including singletons in this analysis has a significant impact on estimated discovery rates at lower sequencing depths (14.8% for contigs, 42.8% for unigenes in the 100K read assembly, Figures S2(a) and S2(c)) but has only a marginal impact for deeper assemblies (70.1% and 83.3% for the 2.2M read assembly). Estimated discovery rates over read lengths and as compared to *B. mori* followed similar trends (Table 1; Additional file 1: Figure S2).

Total percentage of contigs annotated was relatively stable across sequencing depths: 91–94% (94–96%) of perfect contigs (c-isotigs) were annotated compared to *D. melanogaster*, and 60–68% (69–70%) were annotated compared to *B. mori*. These results did not generally trend over sequencing depths, with the exception of *B. mori* annotated perfect contigs, where higher sequencing depth was associated with increasing annotation (not shown). Varying average read lengths, 84–98% (89–96%) of perfect contigs (c-isotigs) were annotated compared to *D. melanogaster*, and 46–80% (57–70%) were annotated compared to *B. mori*. Trends in these percentages were positive as read lengths increased, except for annotations of c-isotigs, which initially increased in percent annotated then decreased slightly as read lengths grew above 400 bp on average (not shown).

Figure 2 revealed that Newbler singletons tend to represent common transcripts. Nevertheless, we still expect many rare transcripts to be represented as singletons alone. To evaluate this possibility, for all sequencing depth tests and for the read length tests where reads average 200 bp or 400 bp (where singleton counts allowed for annotation) we computed the percentages of unique annotations present only in singletons. As expected, the number of transcripts represented only by singletons initially increased then decreased for both assembly types over sequencing depth and decreased by average read length (Table 1, Additional file 1: Figure S3).

As above, for perfect assemblies we can also annotate contigs and singletons based on the transcripts that consituent reads were originally sourced from. In general, for singletons, the number of these unique “true” annotations were between 1.61× and 1.17× higher than the number of unique BLASTX assigned annotations (decreasing with increasing read lengths), reflecting the extent to which BLAST-based annotation assigns paralogous reads identical annotation (Additional file 1: Figure S3; also see Additional file 1: Figure S2 for results on contigs and unigenes).

### Ortholog hit ratio

In O’Neil et al. [4], we introduced a biologically motivated measure of transcript discovery and assembly completeness, known as the Ortholog Hit Ratio (OHR). This measure is defined for each unigene with a BLAST match to a related dataset, and is computed by simply dividing the number of bases in the matched region of the contig by the length of the best-matched sequence [4]. The OHR measure has been used as an estimate of how much of a transcript has been assembled into a contig sequence [4,6,13,14]; ratios close to 1.0 suggest complete transcript assembly.

We computed average and median contig OHRs against both the *B. mori* and *D. melanogaster* protein datasets as sequencing depth increased. For both assembly types OHRs improved with sequencing depth (Table 1, Additional file 1: Figure S4(a)). Average and median OHRs for c-isotigs were larger than for perfect contigs, particularly at lower sequencing depths. This mirrors the earlier result that c-isotigs were fewer but longer. OHRs were generally higher when compared to *D. melanogaster*.

As we increased average read length, OHR statistics increased for the perfect assemblies (Table 1, Additional file 1: Figure S4(b)). At 600 bp when compared to *D. melanogaster*, and at 800 bp when compared to *B. mori*, median OHRs became larger than average OHRs reflecting the contig joining process, bringing many OHR values closer to 1.0. C-isotig OHRs declined with reads longer than 400 bp, consistent with previous results indicating that many reads are left out of the assembly process when reads are long.

As above, including singletons in the analysis improves the use of the OHR metric for assembly evaluation. When computed over contigs and c-isotigs only, c-isotig average and median OHRs were higher than for perfect contigs, whereas over all unigenes these metrics reflect the appropriate relationships and are fully consistent (Table 1, Additional file 1: Figure S4). Due to the large number of singletons produced by the non-perfect assembler (which tend to have similar OHRs given similar read lengths), median statistics were invariant for these assemblies over sequencing depth (Additional file 1: Figure S4).

Because only the matched regions of contigs are used in the OHR measure, untranslated regions on the ends of mRNAs are not included. Further, novel or significantly differentiated genes will lack matches, so overall the OHR measure is designed to be conservative. However, using the OHR as a measure of transcript completeness requires at least two assumptions: first, that the best match indicates an orthologous (rather than a paralogous or erroneous) relationship, and second that the length of orthologous sequence is conserved. While the first assumption can be alleviated by requiring strong match scores (or requiring reciprocal best hits, see below), the second assumption is more problematic. Ortholog hit ratios greater than 1.0 are not uncommon and likely indicate relative expansion of the sequenced transcript relative to the related protein (and/or reduction of the related protein in that species’ evolutionary history). Ratios less than 1.0 could also suffer from such evolutionary inflation or deflation.

**Figure 3:**
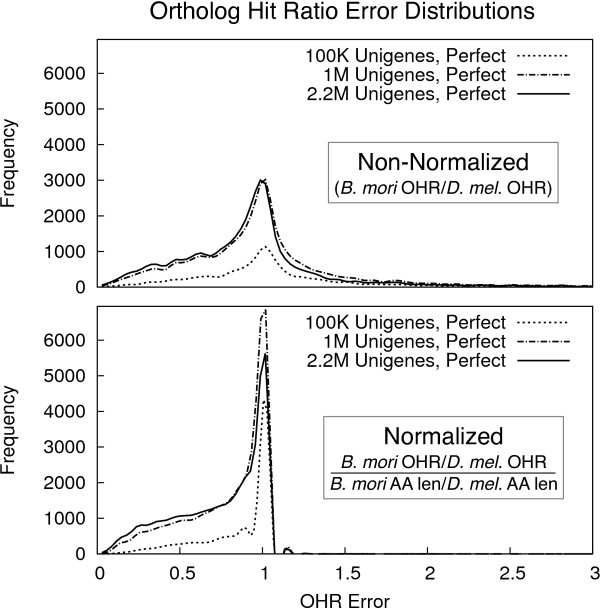
**Ortholog hit ratio error distributions.** Distributions of raw and normalized ortholog hit ratio error, computed as *B. moriOHR*/*D. mel.OHR* and the same ratio divided by the ratio of *B. mori* protein hit length to *D. mel.* protein hit length, for unigenes produced by the perfect assembler given various sequencing depths. Results for other unigene sets (including Newbler-produced unigenes) were similar (not shown).

While these inflations would be impossible to detect in normal circumstances, we computed *D. melanogaster* unigene OHRs against both the *D. melanogaster* protein dataset and the more distantly related *B. mori* protein dataset to investigate this effect. The top panel of Figure 3 shows distributions for the over- or under-estimate of OHR computed as *B. moriOHR*/*D. mel.* OHR, which we call the “OHR Error,” for perfect unigenes (where both OHRs are defined) given various sequencing depths. Overall, we see that the OHR measure is generally conservative: few error ratios are larger than 1.0 and many OHRs are accurate (near 1.0).

To determine whether these errors are inherent to the matching and scoring process, or caused by the relative expansion or contraction of transcripts, we also normalized them by dividing them by the ratio of *B. mori* protein length to *D. melanogaster* protein length. The bottom panel of Figure 3 shows distributions of these normalized ratios: here we see that normalized OHR errors greater than 1.0 are almost entirely absent, indicating that over-estimates in the OHR measure are almost entirely driven by relative expansion in the *D. melanogaster* transcripts. Results varying average read length were similar (not shown).

### Reverse annotation

Just as we can match assembled sequences to *B. mori* and *D. melanogaster* proteins (using BLASTX), we can also do the reverse and match proteins to sequences (using TBLASTN). One common reason for annotating in both directions is to identify sequence and protein pairs that are Reciprocal Best Hits (RBHs). If a sequence and protein have a best match to each other, this is taken as stronger evidence of an orthologous relationship—that the two sequences are descended from the same ancestral locus [29].

Within the limits of sequence comparison, we hypothesized that higher quality assemblies should reveal more RBH relationships, both in absolute terms and as a percentage of BLASTX-annotated sequences. As before, we did not reverse-annotate singletons from non-perfect assemblies of reads averaging longer then 400 bp (due to their large number); because the presense or absense of singletons may affect reverse-annotation rates of contigs as well, we report no data for these assemblies.

As hypothesized, the number of contigs with RBHs against both *B. mori* and *D. melanogaster* followed the same trends as earlier rarefaction results (Table 1, Additional file 1: Figure S5; note that all RBH annotation are by definition unique): RBH count increased with sequencing depth and read length, perfect assemblies result in more RBHs, and comparing to more distantly related proteins results in fewer RBHs (both in absolute count and as a percentage of protein dataset size; Figures S5(a) and S5(b)).

Because RBH counts will naturally increase as the number of annotated sequences increases, we also computed the percentage of BLASTX annotated contigs and c-isotigs that additionally have RBHs, revealing the degree to which increased depth and length allow for increasing rates of high-confidence orthology detection, independent of annotation. In general, percentages of annotated contigs with RBHs were lower in comparison to *B. mori* proteins, and percentages were higher for c-isotigs, reflecting the assembler’s production of fewer but longer c-isotigs which may have been easier to annotate and assign orthology. This trend was reversed when singletons were included: lower percentages of non-perfect unigenes had reciprocal best hits than for perfect unigenes. Otherwise, RBH trends were similar when computed over unigenes (Table 1, Figures S5(c) and S5(d)). In many cases, however, RBH percentages decreased then increased with increasing sequencing depth, preventing this metric from being fully consistent.

**Figure 4:**
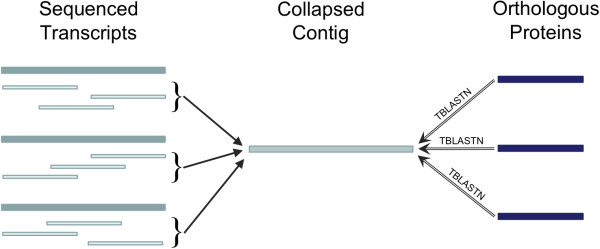
**Possible contig collapse identified in TBLASTN search.** During assembly, a paralogous gene family may be collapsed into a single representative contig. If paralogs are individually represented in a reference dataset, this collapse may be found by matching the related proteins against unigenes and assessing hit counts for target sequences.

Reverse annotation of contigs also revealed those that were matched by more than one protein. These may indicate erroneous “collapse” of reads from similar transcripts into a single consensus sequence (Figure 4). To evaluate this type of error, which should be less prevalent in more accurate assemblies, we assigned each contig and singleton a “Collapse Factor” (CF), computed simply as the number of *D. melanogaster* or *B. mori* proteins having a best match to the sequence.

Table 1 (and Additional file 1: Figure S6) shows trends for average CF computed over contigs (that had a CF of at least 1) as well as over all unigenes (that had a CF of at least 1) against both species as sequencing depth increases. In general, CFs decreased as sequencing depth and read length increased, and are lower when computed via *B. mori*. These lower CFs computed via *B. mori* may reflect the smaller size and relative incompleteness of this dataset; with fewer splice variants and paralogous transcripts correctly identified in this species, fewer proteins share sequence.

Average CFs over c-isotigs were higher than for perfect contigs. Including singletons resulted in average CFs there were very similar between assembly types, though low sequencing depths resulted in average perfect unigene CFs being slightly than average non-perfect unigene CFs (Additional file 1: Figure S6(c)).

Finally, by summing CFs over contigs (or unigenes) we can compute a “reverse rarefaction,” an alternate estimate of transcript discovery based on the number of proteins matching the assemblies. Table 1 shows that these results were similar to the earlier rarefaction results. Here, however, we estimated higher discovery rates. In fact, estimated discovery rates of *D. melanogaster* proteins in perfect contigs and unigenes (Additional file 1: Figure S6) closely reflected the true discovery rates (Additional file 1: Figure S3).

## Discussion

By comparing the results of a perfect assembly process to those of a widely used assembler on a number of simulated datasets, we comprehensively evaluated a host of quality metrics for *de novo* transcriptome assemblies. Our simulated sequencing process presented each assembly type with difficulties unique to transcriptome reconstruction, such as a non-uniform distribution of transcript expression and the presence of alternative splice forms. Recently, a similar simulated sequencing and perfect assembly methodology was used by Mundry et al. to evaluate four assemblers on a single dataset [18]. The results presented here are complementary: by focusing on a single commonly used assembler while varying sequencing depth and average read length, we gain a more in-depth assessment of quality metrics and their response to choice of sequencing technique.

Quality metrics that truly reflect assembly accuracy should reflect the fact that perfect assemblies are by definition of the highest quality. Further, good metrics should also reflect the fact that, for perfect assemblies, increasing sequencing depth or read length produce higher quality output. Although we were not able to annotate singletons for assemblies where they were numerous, these were only found in three of the longest read-length assemblies and were were able to fully annotate two alternate read-length assemblies (200 bp and 400 bp) for all metric assessments. Based on corresponding trends over sequencing depth and for contigs, we do not suspect this limitation affects our conclusions.

Amongst aggregate assembly statistics, we found that simple contig count was not fully consistent with these facts, but metrics influenced by singletons such as percentage of reads assembled and percentage of unigene base pairs in contigs were. Similarly, metrics concerning properties of the output sequences such as average length and coverage were not reliable when computed only over contigs but were fully consistent when computed over all unigenes.

Some annotation-based metrics were fully consistent when computed over both contigs and unigenes, including the number of unique reverse-annotations and the number of reciprocal best hits. Other metrics we considered were never fully consistent: coefficient of variation of read count and percentages of annotated sequences that also had reciprocal best hits. Two annotation-based metrics, average collapse factor and number of unique annotations, were only fully consistent when computed over contigs. This is unsurprising for the average collapse factor, which is primarily a measure of erroneous contig formation. The number of unique annotations in unigenes is inconsistent largely because the number of unique annotations in singletons increases then decreases with sequencing depth.

Of the fully consistent metrics, some provided stronger signals of relative assembly quality than others. For example, although reciprocal best hit count amongst unigenes reflected correct relationships, the counts did not vary greatly over sequencing depth or read length, and counts for perfect assemblies were only slightly higher than for non-perfect assemblies (Figures S5(c) and S5(d)). Further, we noted that while including singletons was necessary to make coverage and length statistics fully consistent, doing so for non-perfect assemblies resulted in coverage and length statistics too invariant to be useful in practice. Stronger metrics included base pairs and percent of base pairs in contigs, unique contig annotations, reciprocal best hit counts, and contig collapse factors. In general, annotation-based metrics appear to reflect assembly quality best when computed over contigs, while basic assembly and sequence statistics should always include singletons.

The ortholog hit ratio, our previously developed annotation-based metric [4], was the exception to the above rule and should be computed over unigenes. Unfortunately, due to the large number of singletons output in non-perfect assemblies, ortholog hit ratios also did not vary strongly over read length or sequencing depth. Previous results, however, indicate that for assemblers outputting fewer singletons (or singleton-reassembly strategies) ortholog hit ratios computed over all unigenes may nevertheless be informative [4]. This may also be the case for the consistent but uniform unigene metrics discussed above.

By comparing *D. melanogaster* unigene OHRs against both *B. mori* and *D. melanogaster* protein sets we discovered that ortholog hit ratios computed against a related species are generally conservative in estimating individual transcript assembly. When the OHR does overestimate the percentage of a transcript assembled, this is almost entirely due to relative expansions in the sequence of interest. While our simulated datasets did not include sequences such as non-coding RNAs or sample contamination (and these may represent confounding artifacts in transcriptome studies [30]), comparison of annotation-based metrics against both *D. melanogaster* and *B. mori* reveals the advantages of having a complete reference for comparison.

Annotation and assessment of source locations for Newbler singletons revealed the difficulties encountered in assembling non-uniformly expressed transcripts. Many singletons were from rare transcripts, as expected, though many more represented highly expressed transcripts. This coupled with the results above suggest that singletons should be considered a proper part of a transcriptome assembly, not only for their biological utility but also in assessing and comparing assemblers and assemblies.

We note that this study does not consider the very high sequencing depths or mated reads provided by technologies such as the Illumina HiSeq platform. While mated reads are expected to provide assembly benefits similar to longer reads, the connection to assembly quality is less clear and so we have not utilized this assumption as we have for read length. Interestingly, under our non-normalized power-law expression model, we found that 2.2 million reads were sufficient to sample of 98% of the true transcriptome when singletons were included. These results suggest additional difficulties for evaluation and annotation of very high coverage depth De-Bruijn based assemblies. For example, while modern assemblers like Trinity [16] can assemble such datasets to a reasonable set of tens of thousands of contigs, significant percentages of reads frequently do not map back, resulting in tens of millions of difficult-to-interpret but potentially important ‘singletons’ (see e.g. [31]).

## Conclusions

Transcriptome sequencing provides researchers with a powerful, cost-effective means of obtaining genetic resources. The uses of a transcriptome assembly are many, and all uses benefit from assembly accuracy. While most papers reporting or comparing transcriptome assemblies also report metrics to speak to their completeness and quality, to date it has been unclear which metrics actually reveal assembly quality in a consistent and identifiable manner. By employing a comparative assembly process using both a “perfect” and an industry-standard assembler while simultaneously varying read lengths and sequencing depths, we comprehensively evaluated a number of quality metrics. While we found some metrics to be accurate measures of assembly quality, others did not—this information will be vital to those working with transcriptomic data, and will ultimately allow researchers to produce useful, comprehensive, cost-effective, and *accurate* genetic resources for nearly any species of interest.

## Methods

### Simulated sequencing

For each assembly, reads were sequenced *in silico* from the *D. melanogaster* transcript set (FlyBase, release 5.32), which includes known transcript splice variants as well as untranslated regions. This dataset contains 23,711 transcripts; these were randomly permuted (the same random permutation was used for all tests). Reads were sequenced randomly from these transcripts according to a power-law distribution modified by read length: in generating read sequences, each transcript *t*_*i*_ in position *i* of the permutation was selected with probability proportional to *P*[*t*_*i*_]∝*l*(*t*_*i*_)/*i* where *l*(·) is the length function, measured in bases. When simulating the sequencing of read *r* from transcript *t*_*i*_, for a given mean read length *l*_*μ*_, a sequence read length *l*(*r*) was selected from min{*l*_*μ*_/4,*N*(*l*_*μ*_,*l*_*μ*_/4)} via resampling (to represent commonly used length cutoffs for read quality filtering). If *l*(*r*)>*l*(*t*_*i*_), we set *l*(*r*)=*l*(*t*_*i*_) and simulated sequencing of the entire transcript. Note that this modification may produce reads shorter than *l*_*μ*_/4 if *l*(*t*_*i*_)<*l*_*μ*_/4, however this only occurred for 732 reads over all simulations (668 of which were for the *l*_*μ*_=1,000 bp simulation). Finally, a contiguous segment of *l*(*r*) bases was selected from transcript *t*_*i*_ uniformly at random. To simulate sequencing error, every base was mutated (uniformly at random to one of the remaining three bases) with probability 0.015.

### Assembly

Although no realistic assembler could be expected to find very short read overlaps, our model of a “perfect” assembler must be able to produce contigs from overlaps shorter than that of the software assembler for our conclusions to be unambiguous. Thus, for perfect assemblies, every set of reads overlapping by one or more bases were used to create contigs (information obtained due to the nature of the simulated sequencing, rather than by comparing reads for overlaps), with consensus sequences being drawn from the original transcripts without sequencing error. Similarly, reads not overlapping other reads (singletons) were error-corrected as part of the assembly output. Non-perfect software assemblies were performed on the command-line with Newbler version 2.5.3, using default options for cDNA assembly: -cdna and -ace. As assemblies were performed on a variety of machines, a variety of CPU (-cpu) numbers were used (between 4 and 12).

### Assembly statistics computation

Assembly statistics (e.g. contig length, coverage, read number) for perfect contigs and singletons were computed during the simulated assembly process.

Newbler information was obtained as follows: singleton sequences were obtained by cross referencing the 454ReadStatus.txt output file with the source reads, while c-isotig sequences were obtained from the 454Isotigs.fna output file. C-isotig statistics were obtained by first converting the 454Isotigs.ace output into AMOS Bank format using the AMOS tools toAmos and bank-transact. Statistics were then obtained using the AMOS tool analyze-read-depth with the -r and -d options. This process produces statistics for a number of c-isotigs that do not appear in the 454Isotigs.fna file (usually representing very short, unused contigs)—only those c-isotigs reported in the 454Isotigs.fna file and singletons reported in the 454ReadStatus.txt file were used for analysis.

Here we note that the assembly process, by default, may split reads for use in multiple c-isotigs. Due to this, the number of reads used per c-isotig as reported from the AMOS Bank over-represents the true number of sequenced reads. For example, for the 600K read/400 bp assembly, we found 645,283 “reads” used in c-isotigs and singletons. Further, some reads are discarded and not reported as singletons. For the 600K read/400 bp assembly, simulated sequencing produced 239.8 Mbp of sequence, whereas summing over coverage ×length for c-isotigs and singletons reveals that only 214.2 Mbp was used in assembly.

### Annotation

Assembled sequences were annotated against both the *Drosophila melanogaster* protein dataset (FlyBase, release 5.38) and the *Bombyx mori* protein dataset (GLEAN produced consensus gene set, SilkDB version 2.0), using BLASTX with a 10^−6^ e-value cutoff keeping only the top match for each query sequence. Similarly, sequences were reverse annotated via these datasets using TBLASTN with a 10^−6^ e-value cutoff keeping only the top match for each query protein.

## Competing interests

The authors declare that they have no competing interests.

## Authors’ contributions

STO designed the methods, performed the analysis, and drafted the manuscript. SE supervised experimental analysis, helped conceive the study, and helped draft the manuscript. Both authors read and approved the final manuscript.

## Supplementary Material

Additional file 1 **Supplementary figures.** An additional PDF file (Additional file 1) providing supplementary figures and corresponding legends describing detailed metric results (Additional file 1: Figures S1 through S6) is available online. Click here for file
