# Supplementary material for: Assessing De Novo transcriptome assembly metrics for consistency and utility
Source: BMC Genomics. 2013 Jul 9;14:465. doi: 10.1186/1471-2164-14-465 (PMC3733778; doi:10.1186/1471-2164-14-465)
Supplement: Additional file 1 — Supplementary figures. An additional PDF file (Additional file 1) providing supplementary figures and corresponding legends describing detailed metric results (Additional file 1: Figures S1 through S6) is available online. [file 1471-2164-14-465-S1.pdf]

Supplementary Materials

Shawn T. O'Neil[1] , Scott J. Emrich*[1]

[1]Department of Computer Science and Engineering, University of Notre Dame, Notre Dame, IN 46556, USA

Email: STO: soneil@nd.edu; SJE*: semrich@nd.edu;

*Corresponding author

Supplementary figures

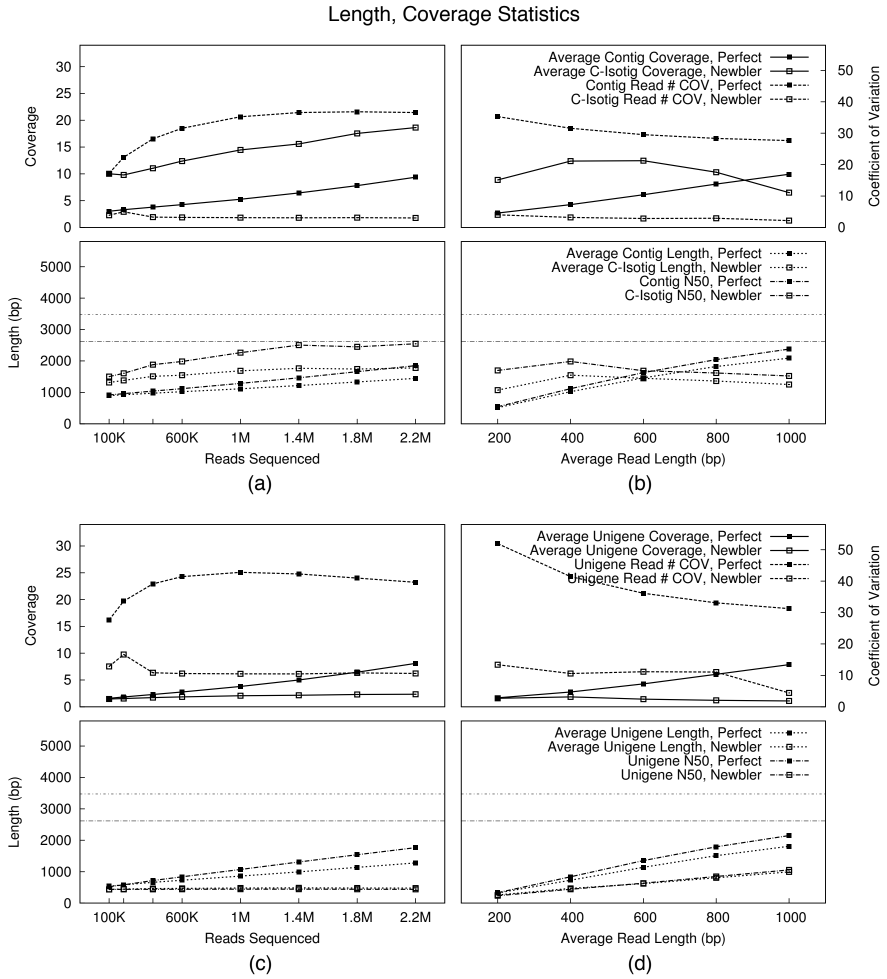

Figure 1: Average sequence coverage and length, read count coefficient of variation, and sequence N50 length as computed over contigs and c-isotigs only ((a) and (b)) as well as over all unigenes (which include singletons; (c) and (d)) as sequencing depth and average read length vary.

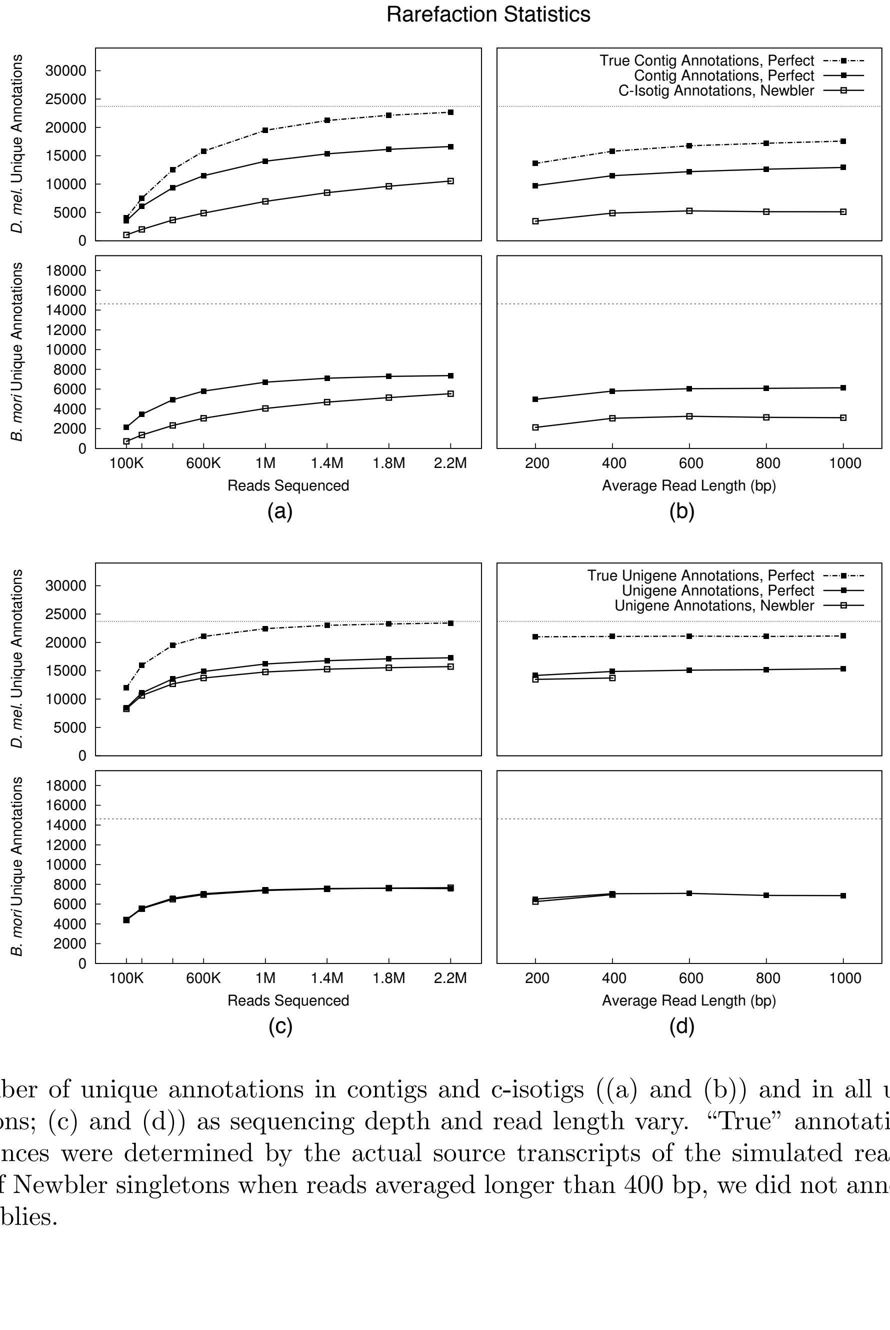

Figure 2: Number of unique annotations in contigs and c-isotigs ((a) and (b)) and in all unigenes (which include singletons; (c) and (d)) as sequencing depth and read length vary. "True" annotations for perfect-assembly sequences were determined by the actual source transcripts of the simulated reads. Due to the large number of Newbler singletons when reads averaged longer than 400 bp, we did not annotate singletons for these assemblies.

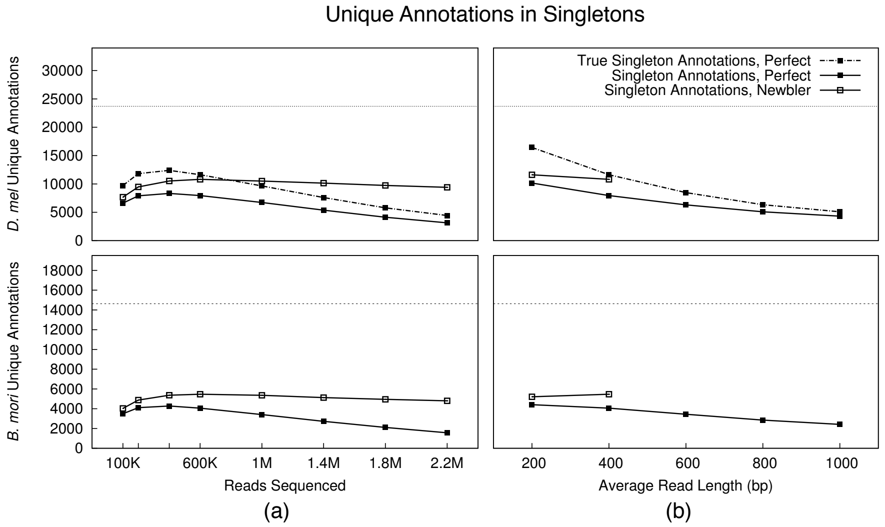

Figure 3: Number of unique annotations found only in singleton sequences as sequencing depth and read length vary. "True" annotations for perfect-assembly sequences were determined by the actual source transcripts of the simulated reads. Due to the large number of Newbler singletons when reads averaged longer than 400 bp, we did not annotate singletons for these assemblies.

Ortholog Hit Ratio Statistics

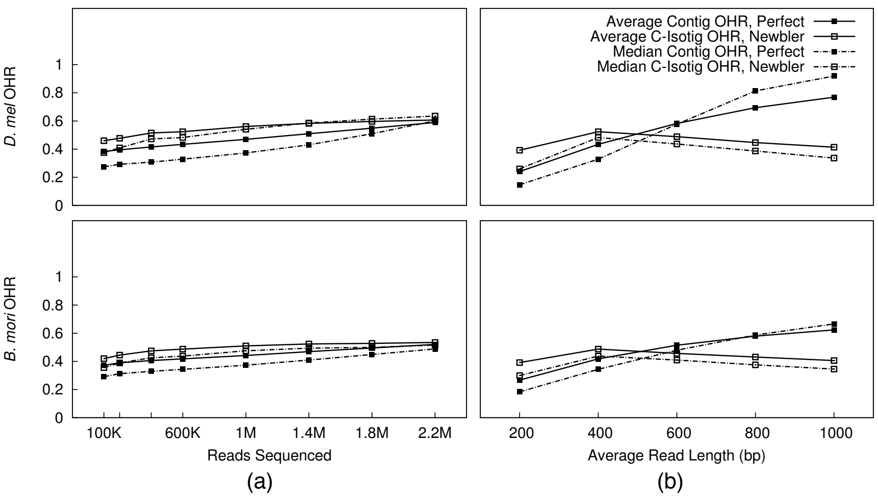

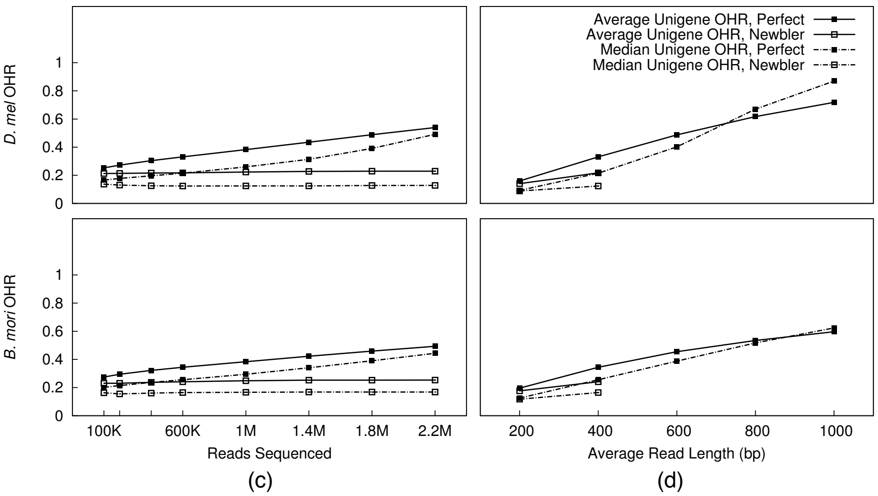

Figure 4: Average and median ortholog hit ratios over contigs and c-isotigs ((a) and (b)) and all unigenes (which include singletons; (c) and (d)) as sequencing depth and read length vary. Due to the large number of Newbler singletons when reads averaged longer than 400 bp, we did not annotate singletons for these assemblies.

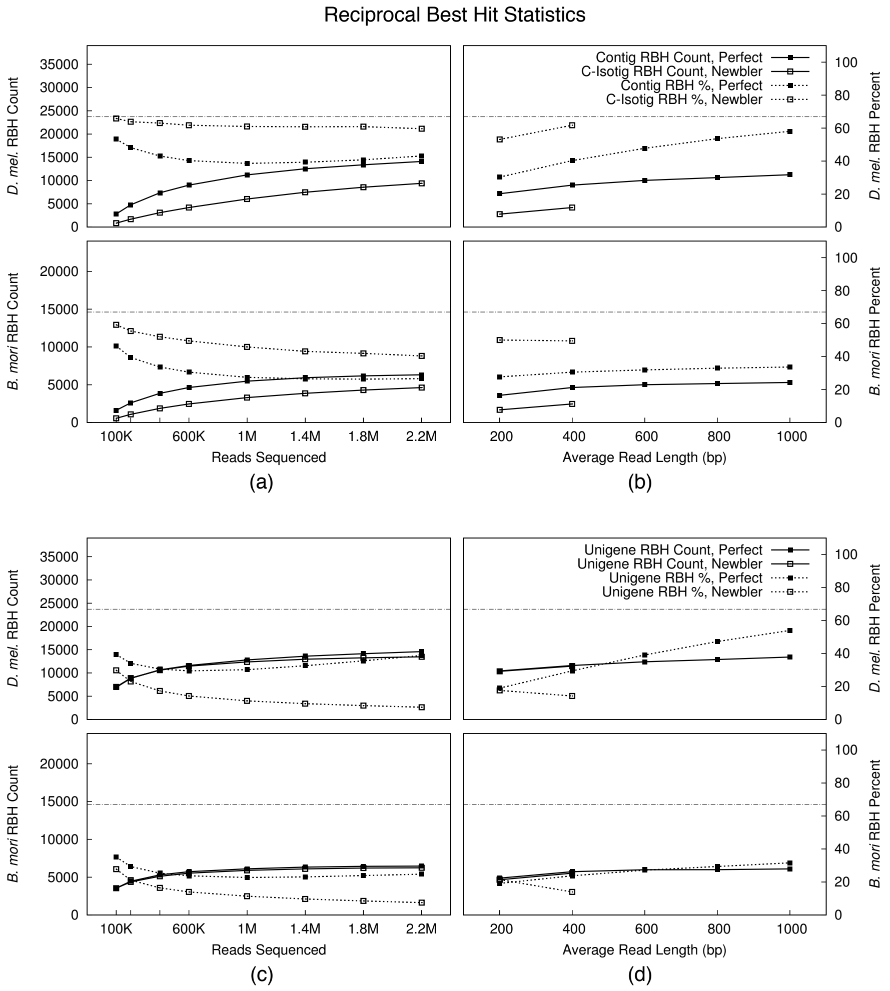

Figure 5: Counts of reciprocal best hit annotations, as well as percentages of BLASTX annotated sequences additionally having reciprocal best hits, for contigs and c-isotigs ((a) and (b)) and all unigenes (which include singletons; (c) and (d)). Due to the large number of Newbler singletons when reads averaged longer than 400 bp, we did not reverse-annotate (using TBLASTN) these assemblies.

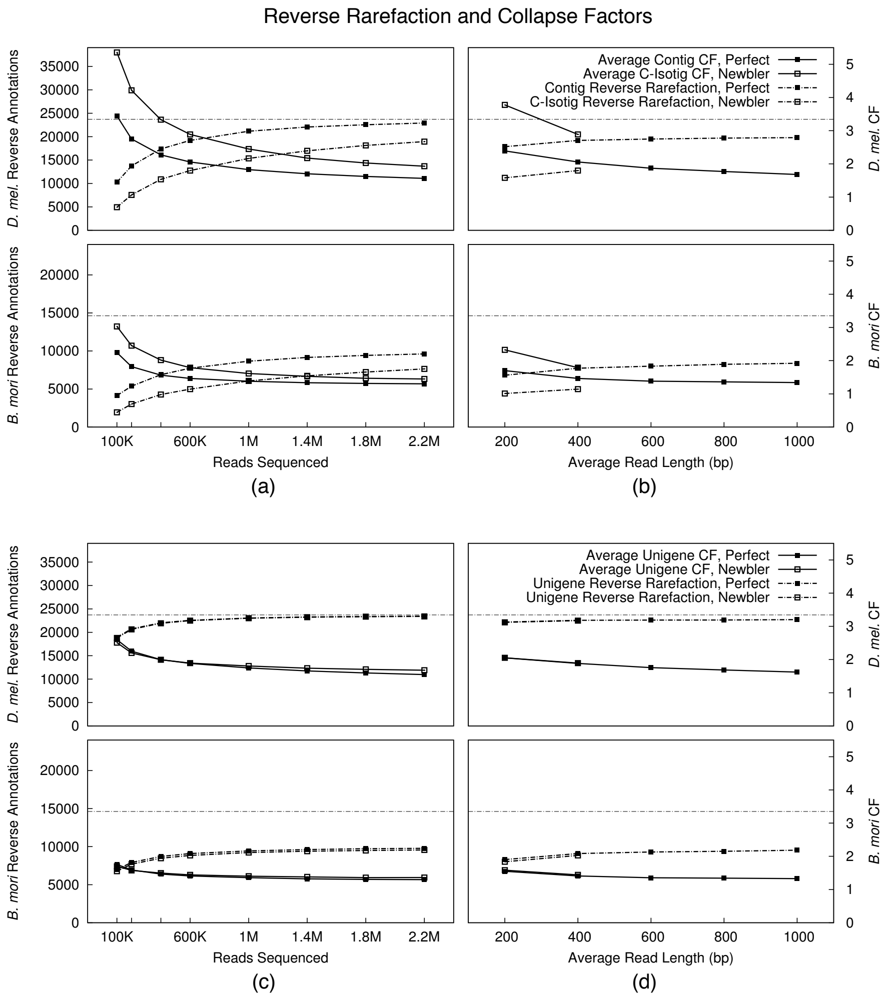

Figure 6: Counts of (necessarily unique) reverse-annotations and average collapse factor (amongst sequences with at least one reverse-annotation) over contigs and c-isotigs ((a) and (b)) as well as over all unigenes (which include singletons; (c) and (d)). Due to the large number of Newbler singletons when reads averaged longer than 400 bp, we did not reverse-annotate (using TBLASTN) these assemblies.
